# Solving Patient Allocation Problem during an Epidemic Dengue Fever Outbreak by Mathematical Modelling

**DOI:** 10.3390/healthcare10010163

**Published:** 2022-01-15

**Authors:** Jung-Fa Tsai, Tai-Lin Chu, Edgar Hernan Cuevas Brun, Ming-Hua Lin

**Affiliations:** 1 Department of Business Management, National Taipei University of Technology, Taipei 10608, Taiwan; jftsai@ntut.edu.tw (J.-F.T.); tlchu@mail.ntut.edu.tw (T.-L.C.); hernancbrun@gmail.com (E.H.C.B.); 2 Department of Urban Industrial Management and Marketing, University of Taipei, Taipei 11153, Taiwan

**Keywords:** dengue fever, epidemic, optimization, patient allocation, mathematical techniques

## Abstract

Dengue fever is a mosquito-borne disease that has rapidly spread throughout the last few decades. Most preventive mechanisms to deal with the disease focus on the eradication of the vector mosquito and vaccination campaigns. However, appropriate mechanisms of response are indispensable to face the consequent events when an outbreak takes place. This study applied single and multiple objective linear programming models to optimize the allocation of patients and additional resources during an epidemic dengue fever outbreak, minimizing the summation of the distance travelled by all patients. An empirical study was set in Ciudad del Este, Paraguay. Data provided by a privately run health insurance cooperative was used to verify the applicability of the models in this study. The results can be used by analysts and decision makers to solve patient allocation problems for providing essential medical care during an epidemic dengue fever outbreak.

## 1. Introduction

Dengue fever is considered the most rapidly transmitted arboviral disease in recent years [1]. It is mainly transmitted by two species of female mosquitoes, Aedes aegypti and Aedes albopictus [2]. Four distinctive virus serotypes can cause dengue fever: DEN-1, DEN-2, DEN-3, and DEN-4 [3]. Originally, the dengue virus was only recurrent in tropical and sub-tropical regions, but today it can be encountered in 128 countries, including the nations in the European and North American continents, both of which are predominantly non-tropical regions. Recent studies estimate that 3.9 billion people are at risk of being infected every year. It has also been reported that 390 million dengue infections occur every year, resulting in 500,000 hospitalizations and 20,000 deaths due to severe complications [4]. Besides adopting conventional preventive measurements to combat the vector mosquitoes and surveillance practices [5], the World Health Organization also recommends the application of vaccines, especially in areas that are described as highly endemic [3].

The economic burden caused by dengue fever epidemics has considerably impacted many countries. In the Americas, the aggregate annual cost, combining ambulatory and hospitalized cases, reached US$ 2.1 billion during the period 2000–2007 [6]. In order to counteract these losses, a more effective allocation of patients and medical resources is required along with well-established health policy priorities and the implementation of disease control technologies [7].

In recent years, many optimization methods have been implemented to solve problems involving the allocation of patients and medical resources, such as scheduling of operation rooms, allocation of emergency patients and allocation of healthcare facilities. Burdett et al. [8] discussed a mixed linear programming approach to assess hospital resources and capacity. Their method aimed to help hospitals with diverse capacity planning and resource allocation activities. Zhou et al. [9] presented another multi-objective model that focused on the allocation of hospital wards considering revenue and equity factors. The objective of the stochastic programming model was to maximize both factors considered in the model. For operating room scheduling, Molina-Pariente et al. [10] presented a mixed integer linear programming model that solved problems integrating operating room planning and scheduling with surgical teams based on the surgeons’ experience and skills. Beroule et al. [11] introduced a series of meta-heuristic methods to resolve a more subject-specific operating room scheduling that dealt with medical device sterilization.

A more extensive study conducted by Steiner et al. [12] covered a case in Parana, Brazil that aimed to improve the existing healthcare system in the region. The multi-objective approach was set to maximize population homogeneity in designated microregions within the state, to maximize the variety of medical procedures offered in each area, and to minimize the distance travelled by patients between regions. Heshmat et al. [13] used clustering and mathematical programming to solve the scheduling of appointments in outpatient chemotherapy clinics.

Studies on the management of allocation of emergency medical services have also been conducted. Shahriari et al. [14] presented a model that minimized the total time of casualties being transferred from an incident spot to a medical facility, considering the uncertainties of the demand points. Anparasan and Lejeune [15] proposed an integer linear programming model to determine the number, size, and location of treatment facilities, deploy critical medical staff, locate ambulances to triage points, and organize the transportation of severely ill patients. They also developed an algorithmic procedure using hierarchical constraints and valid inequalities to reduce the solution time. Devi et al. [16] proposed a mixed-integer linear programming model to design the topology of health care facility networks. Their model considers minimizing the total cost of establishing and operating temporary testing laboratories and minimizing the maximum travel time from a patient node to a testing facility. Esra Büyüktahtakın et al. [17] introduced an epidemics–logistics mixed-integer programming model to determine the optimal amount, timing and location of resources. Their model aims to minimize the total number of infections and fatalities under a limited budget over a multi-period planning horizon. Liu et al. [18] modified the Esra Büyüktahtakın et al. [17] model by changing capacity constraint, and then applied it to control the 2009 H1N1 outbreak in China. Liu et al. [18] formulated the problem as a mixed-integer non-linear programming model and determined when to open the new isolated wards and when to close the unused isolated wards.

Recently, the rapid outbreak of the coronavirus has attracted increasing attentions from researchers to apply mathematical techniques for solving resource allocation problems in healthcare system. Different mathematical models are constructed by [19,20,21,22,23,24,25] to optimally allocate critical hospital supplies to treat COVID-19 patients. For instances, [22,23] considered an optimal allocation of ICU beds (intensive care unit beds) and personal protective equipment, respectively, under the scarcity of available resources. A binary integer optimization model is developed to find the best allocation for ICU beds, considering candidate patients with suspected/confirmed COVID-19 [22]. Santini [24] proposed an Integer Programming formulation to maximize the number of tests a country can perform and validated their approach on both real-life data from Italy and synthetic instances. Jordan et al. [26] provided a comprehensive review of optimization in the context of COVID-19 prediction and control.

Additionally, the Internet of Things (IoT) can create innovative applications in healthcare. IoT-based real-time patient-generated medical data that can be collected from wearable networked sensor-based devices are used to predict and control infectious diseases [27,28,29]. During the COVID-19 pandemic, healthcare providers in the areas where dengue is endemic or who are treating patients with recent travel history to the coronavirus pandemic areas need to consider dengue and COVID-19 in the differential diagnosis of acute febrile illnesses. Several studies [30,31,32] focused on stress-related psychiatric disorders, clinically significant depression, and elevated anxiety symptoms among medical personnel providing care to COVID-19 patients.

The extension of allocation models can be generally classified into long-term, short-term, and medium-term based on the entire scheduling time period considered in the optimization approaches. Currently, the literature concerning medium-term planning allocation models is more limited. Medium-term planning models cope with situations that take longer than a few days, but less than years. Epidemics and pandemics fall into this category. There are many diseases that can cause these events, for examples, influenza, dengue fever, chikungunya, and yellow fever. Patient and resource allocation among medical facilities during a pandemic influenza outbreak was investigated by Sun et al. [33].

Epidemic and pandemic events, topics treated in this paper, have become more recurrent in the past two centuries. An increase in global population, surpassing 7 billion inhabitants, and the fast speed of globalization have contributed to the increase in infectious diseases, not to mention the effect of natural disasters, human conflicts, and high levels of poverty in some areas. Fortunately, advances in technology and science have forged a new path for the analysis and response toward these events. Today, in the 21st century, the systematical combination of modern epidemiology, new field-based rapid diagnostic tools, vaccinations, clinical care standards, communication, and real-time training allow to provide a more efficient response to the reappearance of old threats, as well as the introduction of new ones [34].

Paraguay is a high-endemicity country for the dengue disease. Since 2009, dengue fever has emerged as a critical health problem in Paraguay with periodic significant epidemic outbreaks [35,36]. Due to the dengue epidemic, which has a significantly negative impact on population health and economic development in Paraguay, it is necessary to develop appropriate mechanisms of response to face the dengue outbreak. One of the major issues is how to allocate infected patients in different hospitals to receive appropriate care. Although mathematical modelling is an effective technique for resource allocation, few studies in the literature focused on patient allocation problem in a dengue outbreak in Paraguay. Therefore, this study intends to apply mathematical modelling for solving this problem in Paraguay that have not been intensively treated in the past. The aim of this study is to provide an effective mechanism for the management of medical facilities and resources during a dengue fever epidemic. The models intended to help decision makers to order and distribute additional resources for the most appropriate areas, optimizing the occupancy of hospitals, the usage of medical resources, and the working hours of medical personnel. By combining the factors mentioned above, three models were utilized to analyze a dengue fever epidemic.

The rest of the paper is organized as follows. Section 2 describes the case of a privately run health insurance cooperative in Ciudad del Este, Paraguay and defines the three mathematical models used in this paper. In Section 3, an analysis of the empirical results is summarized to present the most relevant findings of this paper. Section 4 presents a discussion and clarifies the practical contributions of this paper. The final section, Section 5, contains the conclusions and some important remarks.

## 2. Materials and Methods

**Figure 1:**
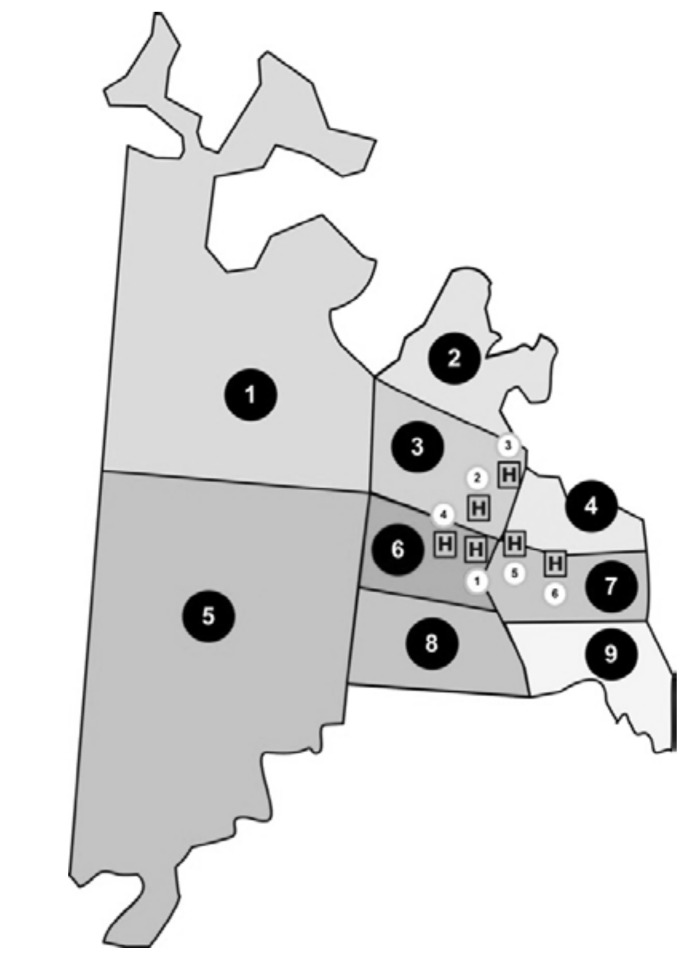
Population areas and hospitals in the map of Ciudad del Este.

Study area. Ciudad del Este is a city located on the east side of the Paraguay Oriental Region. The empirical study in this paper was performed using data provided by a privately run health care cooperative in Ciudad del Este. A total of six hospitals located within the territory of Ciudad del Este are considered in this study. In 2018, the estimated population was 299,255 inhabitants [37]. The total area of Ciudad del Este can be divided into nine sections based on an estimated number of inhabitants per section. Figure 1 indicates the nine population areas along with the location of six hospitals that provided medical coverage to approximately 37,000 people under the health care cooperative. The distances between the polygon centroids of the nine population areas and six hospitals are shown in Table 1.

The numbers of non-ICU beds, ICU beds, and doctors and nurses available during epidemics in the six hospitals are listed in Table 2. Nurses took rounds of 12 h a day, while doctors’ rounds per day were extended throughout 24 h. Two types of patients were taken into consideration: type 1 (P1) patients admitted into non-ICU beds, requiring 15 min of medical assistance provided by doctors and 30 min of assistance provided by nurses, and type 2 (P2) patients hospitalized in ICU beds that required 15 min of assistance provided by doctors and 40 min by nurses per day. The length of stay for P1 and P2 patients was 3 and 6 days, respectively. Table 3 and Table 4 list the estimated demand data per population area during six weeks of a dengue fever epidemic in Ciudad del Este, Paraguay. The estimated demand is based on the historical data of the number of patients suffering from dengue fever in the areas most affected by the epidemic. The demand of non-ICU beds will be higher in areas A2, A3, and A5 than other areas. The demand of ICU beds will be higher in areas A2, A5, and A9 than other areas. Both demands of non-ICU beds and ICU beds will reach a high level in week 4.

The methodology presented in this study was based on algorithms formulated by Sun et al. [33], using concepts of linear programming.

### 2.1. Model 1–Single Objective Function and Planning Horizon

Model 1 allocates patients based on a single objective function that minimizes the total distance travelled by all patients to reduce cost. It involves a single planning horizon, also referred to as the current planning horizon. The first requirement of the model is to define the distance between each population area and hospital. The demand of patients of each type is also established along with the resources that the patient needs and the length of stay required before the patient is discharged. The initial capacity of each resource is recorded at the beginning of the current planning horizon to oversee its future availability. Patients who are hospitalized before the current planning horizon and released patients who are admitted in previous planning horizons are further considered in the model for potential implications related to occupancy.

This model does not consider the medical personnel involved in the hospitalization and treatment process; it only deals with material resources. The parameters and variables used in this study are introduced as follows.

Sets

*A*: set of population areas
*H*: set of hospitals
*P*: set of patient types
*R*: set of medical equipment types
*T*: set of time periods in the current planning horizon
*S_r_*: set of patient types requiring medical equipment type *r*

Parameters

*Distance_a,h_*: distance from population area *a* to hospital *h*
*Demand_p,a,t_*: demand of patient type *p* from population area *a* on day *t*
*IniCapacity_r,h_*: initial capacity of medical resource *r* in hospital *h*
*LOS_p_*: length of stay of patient type *p*
*InitialPatients_p,h_*: number of patients of type *p* at hospital *h* from previous planning horizon
*ReleasedPatients_p,h,t_*: number of patients of type *p* at hospital *h* from previous planning horizon who are discharged on day *t*

Variables

*NoPatients_p,h,t_*: number of patients of type *p* at hospital *h* on day *t*
*X_p,a,h,t_*: number of patients of type *p* from population area *a* assigned to hospital *h* on day *t*

The patient allocation optimization problem, referring from Sun et al. [33], can be formulated as follows.

Model 1:

(1) $$\min\;\sum_{p\in P}\sum_{a\in A}\sum_{h\in H}\sum_{t\in T}X_{p,a,h,t}\times Distance_{a,h},$$

subject to:

(2) $$\sum_{h\in H}X_{p,a,h,t}=Demand_{p,a,t}\;\forall \;p\in P,\;\forall a\in A,\;\forall t\in T,$$

(3) $$NoPatients_{p,h,1}=InitialPatients_{p,h}+\sum_{a\in A}X_{p,a,h,1}\;\forall \;p\in P,\;\forall h\in H,$$

(4) $$NoPatients_{p,h,t}=NoPatients_{p,h,t-1}+\sum_{a\in A}X_{p,a,h,t}-\sum_{a\in A}(X_{p,a,h,t-LOS_{p}})-ReleasedPatients_{p,h,t}\;\forall \;p\in P,\;\forall h\in H,\;\forall t\in T,\;t>1,$$

(5) $$\sum_{p\in S_{r}}NoPatients_{p,h,t}\leq IniCapacity_{r,h\;}\;\forall \;r\in R,\;\forall h\in H,\;\forall t\in T,$$

(6) $$X_{p,a,h,t}\geq 0\;\forall \;p\in P,\;\forall a\in A,\;\forall h\in H,\;\forall t\in T.$$

The objective function (1) aims to minimize the total distance travelled by all patients to a hospital. Constraint (2) deals with the demand of patients, ensuring that every patient is admitted into a hospital. Constraints (3) and (4) are related to the intermediate variable for the number of patients, assuming conditions on the first day and the following days, respectively. Constraint (5) ensures the availability of resources, and constraint (6) specifies that the decision variables have to be greater than or equal to zero.

### 2.2. Model 2–Multiple Objective Function and Planning Horizon Including Medical Personnel Working Hours

Model 2 introduces a bi-objective function and multiple planning horizons to enhance capabilities and flexibility. A second objective that minimizes the total distance travelled by each patient is incorporated. Moreover, considering multiple planning horizons in Model 2 that can define the length of periods and accept data modification is more applicable to epidemics, especially because the scenarios during these events may vary radically from one day to another. The additional parameters and variables are introduced as follows.

Additional Sets

*T*′: set of time periods on the next planning horizon
*S*: set of medical personnel types (i.e., doctor, nurses)
*S_s_*: set of patient types requiring the assistance of medical personnel type *s*

Additional Parameters

*StaffHours_p,s_*: staff hours required for patient type *p* of staff type *s*
*StaffCapacity_s,h,t_*: staff capacity in hours of staff type *s* at hospital *h* on day *t*
*ResourceShortage_r,t_*: shortage of medical resource *r* on day *t*
*StaffHrsShortage_s,t_*: shortage of staff hours *s* on day *t*
*M*: A large number

Additional Variables

*ReleasedPatients_p,h,t_*: number of patients of type *p* who are admitted in a previous planning horizon and will be released from hospital *h* on day *t* of the current planning horizon
*NextReleasedPatients_p,h,t_*: number of patients of type *p* who are admitted in current planning horizon but will be released from hospital *h* on day *t*′ of the next planning horizon
*Y_a,h_*: equals 1 if patients are admitted in hospital *h*, otherwise 0
*D*: maximum distance from patient to assigned hospital

The bi-objective function is

(7) $$\left\{\mathrm{Minimize}\;\sum_{p}\sum_{a}\sum_{h}\sum_{t}X_{p,a,h,t}\times Distance_{a,h}\;,\;\mathrm{Min}\;D\right\},$$

By adding the following constraints (12)~(14), the second objective in objective function (7) is considered in the constraint set. After transforming the bi-objective function into a single objective function, the patient allocation optimization problem that minimizes the total distance travelled by each patient factoring in multiple planning horizons, referring from Sun et al. [33], can be formulated as follows.

Model 2:

(8) $$\min\;\sum_{p\in P}\sum_{a\in A}\sum_{h\in H}\sum_{t\in T}X_{p,a,h,t}\times Distance_{a,h}+\sum_{p\in P}\sum_{a\in A}\sum_{t\in T}X_{p,a,h^{\prime },t}\times Distance_{a,h^{\prime }}\times \left(T+1-t\right),$$

subject to:

(2) ~ (6)

(9) $$NoPatients_{p,h,1}=InitialPatients_{p,h}+\sum_{a\in A}X_{p,a,h,1}-ReleasedPatients_{p,h,1}\;\forall p\in P,\;h\in H,$$

(10) $$NextReleasedPatients_{p,h,t^{\prime }}=\sum_{a\in A}X_{p,a,h,t^{\prime }+T-LOS_{p}}\;\forall p\in P,\;h\in H,\;t^{\prime }\in T^{\prime },$$

(11) $$\sum_{p\in S_{s}}\left(NoPatients_{p,h,t}\times StaffHours_{p,s}\right)\leq StaffCapacity_{s,h,t}\;\forall \;s\in S,\;h\in H,\;t\in T,$$

(12) $$Distance_{a,h}Y_{a,h}\leq D\;\;\forall a\in A,\;h\in H,$$

(13) $$\sum_{p\in P}\sum_{t\in T}X_{p,a,h,t}\leq M\times Y_{a,h}\;\;\forall a\in A,\;h\in H,$$

(14) $$Y_{a,h}=0\;\mathrm{or}\;1\;\;\forall a\in A,\;h\in H,$$

(15) $$\sum_{p\in S_{r}}NoPatients_{p,h^{\prime },t}\;\;\forall \;r\in R,\;t\in T,$$

(16) $$StaffHrsShortage_{s,t}=StaffCapacity_{s,h,t}-\sum_{h\in H}\sum_{p\in S_{s}}NoPatients_{p,h,t}\times StaffHours_{p,s}\;-\sum_{p\in S_{s}}NoPatients_{p,h^{\prime },t}\times StaffHours_{p,s}.$$

Model 2 is based on a bi-objective function presented in (7). Equation (8) presents a single objective function transformed from (7). In order to increase the flexibility of the algorithm, a dummy hospital h’ is introduced for dealing with the patients who may be rejected due to the lack of enough resources. The second set of summations referred to the dummy hospital where the expression $\left(T+1-t\right)$ ensures that patients were assigned to the dummy hospital as the last way to resolve the model, minimizing the second objective. Constraint (10) involves the number of patients who will be released in the next planning horizon. Medical personnel capacity is referred to in constraint (11). Constraints (12)–(14) are the constraints related to the second objective. Equations (15) and (16) are incorporated to predict when resource shortage and shortage of staff hours occur.

### 2.3. Model 3–Multiple Objective Function and Planning Horizon Including Additional Resources

Model 3 investigates the advantage of allocating additional resources of all types. Using the improvements made in Model 2, Model 3 optimizes the model considering additional medical resources and medical personnel working hours. The additional parameters and variables are introduced as follows.

Additional Parameters

*AddResourcesr*,*t*: number of additional resource *r* available on day *t*
*AddPersResourcess*,*t*: working hours of additional personnel resource *s* on day *t*

Additional Variables:

*AddRAllocationr*,*h*,*t*: number of resource *r* allocated to hospital *h* on day *t*
*AddPRAllocations*,*h*,*t*: working hours of medical personnel type *s* allocated to hospital *h* on day *t*

The patient allocation optimization problem that minimizes the total distance travelled by each patient considering in multiple planning horizons and considers additional medical resources and medical personnel working hours, referring from Sun et al. [33], can be formulated as follows.

Model 3:

(8) $$\min\;\sum_{p\in P}\sum_{a\in A}\sum_{h\in H}\sum_{t\in T}X_{p,a,h,t}\times Distance_{a,h}+\sum_{p\in P}\sum_{a\in A}\sum_{t\in T}X_{p,a,h^{\prime },t}\times Distance_{a,h^{\prime }}\times \left(T+1-t\right),$$

subject to:

$$\left(2\right)\sim \left(6\right),\;\left(9\right)\sim \left(16\right)$$

(17) $$\sum_{h\in H}AddRAllocation_{r,h,t}\leq AddResources_{r,t}\;\;\;\forall \;r\in R,\;t\in T,$$

(18) $$\sum_{h\in H}AddPRAllocation_{s,h,t}\leq AddPersResources_{s,t}\;\;\;\forall \;s\in S,\;t\in T.$$

Constraints (17) and (18) ensure that the allocation of additional medical resources and additional working hours of medical personnel, respectively, does not exceed the available additional capacity. Model 3 can help decision makers identify areas where these resources could be allocated.

## 3. Results

This study used the data provided by a privately run health care cooperative in Ciudad del Este to investigate the patient allocation optimization problem. In order to provide an efficient response to the outbreak, all beds in each hospital shown in Table 2 were assumed to be available. Data belonging to week 1 was set as the only input for Model 1. Table 5 shows the allocation of patients from the results of Model 1. More than one value in a box meant patients were allocated in more than one hospital. The objective function value was 323.08, accounting for 125 patients.

In order to solve the multiple objective model, the ε-constraint method was applied. In this study, an upper limit with respect to patient travel distance to a hospital was set to 9. The total patient travel distance was identified as 7.11 km and remained unchanged when the upper limit was larger than 7.5 km. However, the solution became infeasible when the upper limit was fixed at 7 km or below. The results from Model 1 and Model 2 were mostly similar for week one. Week 2 represented the second planning horizon in Model 2. The values from $NextReleasedPatients_{p,h,t^{\prime }}$ in week 1 were used as $ReleasedPatients_{p,h,t}$ in week 2, and the $InitialPatients_{p,h}$ values were also based on the results of week 1. At the end of week 2, no medical resources were exhausted, and all patients were allocated to existing medical facilities. The objective function value stood at 419.41 for a total number of 156 patients.

As the number of patients seeking medical assistance continued to grow, week 3 was identified as the first week in which medical resources were not sufficient. Table 6 shows the allocation of patients during week 3.

Resource shortage was observed when patients were allocated in the dummy hospital represented by H7. The model showed that there was no medical personnel shortage, but there was a shortage of both non-ICU and ICU beds. Table 7 shows the medical resource shortage during week 3 obtained from constraints (15) and (16).

Similarly, a variation on medical personnel availability was involved to demonstrate personnel resource shortage. Previous results were performed using the entire number of medical personnel hours. Data from week 1 was further analyzed considering availability of 50%, 30%, 10%, 9%, and 8%. Results remained unchanged at availability of 50% and 30%; however, when only 10% and 9% of all available hours were considered, the optimal solution increased to 329.59. When the staff capacity was lowered to 8%, the solution became infeasible.

From the results of Model 2, the existing medical equipment was insufficient to satisfy the demand of new admissions during week 3. Table 7 shows that the maximum shortage for non-ICU beds is 34 and the maximum shortage for ICU beds is 23 during week 3. By using these values, Model 3 distributed additional resources such that all patients were successfully allocated in the existing hospitals. The distribution of additional resources is displayed in Table 8. The total numbers of additional non-ICU beds and ICU beds that can cover the shortage listed in Table 7 are 34 and 23, respectively. Model 3 allocated the additional resources in appropriate hospitals. The objective function value for week 3 was 663.11.

After allocating the newly available medical resources listed in Table 8, the results of week 4 obtained from Model 3 showed that another medical resource shortage event occurred. However, the medical personnel working hours were sufficient. Model 3 was used to find the optimal distribution of resources and to rearrange the allocation of patients in the existing hospitals. The model reallocated the patients and produced an objective value of 681.33 for all 251 patients. As the last two weeks proceeded, the number of admissions per day continued to decrease. Therefore, the available resources were able to cover all the requirements.

## 4. Discussion

**Figure 2:**
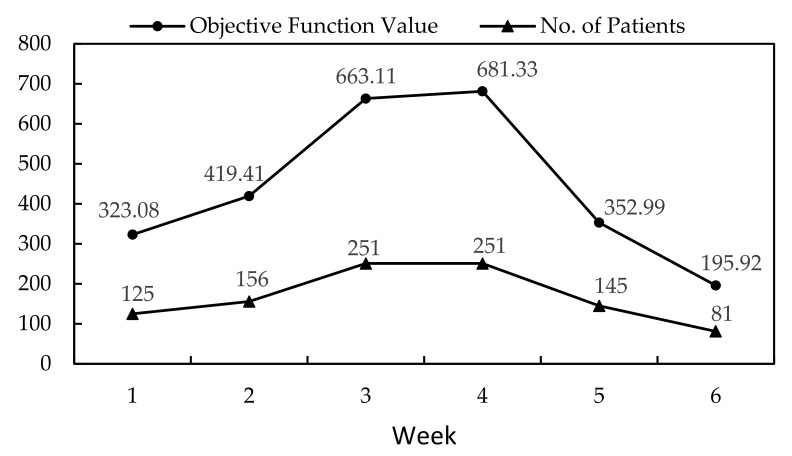
Objective function values (in kilometers) and number of patients during the six-week period.

The case study in this paper served as an example on how the course of an epidemic could be treated. Figure 2 illustrates the objective function values (the distance travelled by all patients) obtained from Model 2 and Model 3, and the number of patients admitted throughout the 6 weeks. The six-week patient data is almost symmetric distribution with the peak number 251 of the outbreak occurred during weeks 3 and 4. We observed that objective function value (in kilometers) increased as the number of patients increased. Both objective function value and the number of patients decreased after the peak, leading to lower points at the end of week 6.

Figure 2 indicates that the number of patients reaches a highest level in week 3 and week 4. According to Table 3 and Table 4, the estimated demands of ICU and non-ICU beds during six weeks increase gradually at first and reach a high level in week 4. If the medical resources are not enough to take care infected people, the dengue fever epidemic becomes severe and the number of patients in hospitals remains high for a long time due to medical resource shortage. Then people require a longer time to return to pre-pandemic normalcy. Since Model 3 suggests how to allocate additional resources to provide enough medical services for patients, the number of patients in the hospitals goes down gradually after the number of infected people reaches a high level.

This study exemplified that resource allocation is a critical factor to attain a better organizational structure. The results of the models provided a comprehensive understanding of the distribution of the equipment, facilities and medical personnel for healthcare organizations and analysts. The practical contributions of this study are listed as follows:

- To reduce costs for patients who need to travel to medical facilities, therefore reducing the time for patients to be admitted to wards and decreasing the probability of virus spread.
- To distribute additional medical resources (i.e., non-ICU beds and ICU beds) throughout different hospitals considering the development of an epidemic, therefore avoiding the shortage of medical resource and the expansion of epidemic outbreaks.
- To stipulate the period and place where a resource could be exceeded, therefore developing a response plan in advance.
- To analyze the process of an outbreak by presenting multiple planning horizons, thus enabling to adjust the allocation of patients and additional resources more effectively.

## 5. Conclusions

It has been reported that appropriate levels of preparedness are crucial to deal with the rapid spread of dengue fever epidemic; however, in many occasions, it is difficult to predict their evolution, which easily leads to a shortage of equipment and human resources. The study adopts mathematical modelling to optimize the allocation of patients and additional resources during an epidemic caused by diverse sources. Previous works presented similar ideas for allocation, but few studies in the literature focused on using mathematical modelling to solve patient allocation problem in a dengue outbreak in Paraguay. This study applies single and multiple objective linear programming models to optimally allocate patients and additional resources in hospitals during an epidemic dengue fever outbreak, minimizing the total distance travelled by all patients. Based on the analytical results, the decision makers can allocate patients in the most appropriate hospital and distribute additional medical resources to avoid the shortage of medical resource and the expansion of epidemic outbreaks.

For the patient allocation problem during a pandemic outbreak, there are still several future research directions. Existing models can be extended to consider the capability of hospital. The hospitals can be classified into several categories to take care of patients in different phases of infection. Some hospitals are more likely to admit the severely injured patients and others are responsible for treating slightly injured patients. Additionally, the implementation of IoT-based technologies can substantially collect real-time patient-generated medical data to predict the transmission of infectious diseases. The correct prediction of future cases of infectious disease is helpful in preparing more medical resources earlier to take care the rapid growth of dengue cases and mitigate the ramifications of a dengue pandemic. These issues are important and worthy of future investigation.

## Figures and Tables

**Table 1:** Distances between areas and hospitals in kilometers.

Area	Hospital					
	H1	H2	H3	H4	H5	H6
A1	7.37	6.89	7.19	6.80	8.08	9.19
A2	4.99	3.99	3.43	4.93	4.99	5.82
A3	3.19	2.30	2.28	2.95	3.50	4.52
A4	2.56	2.17	1.56	3.18	1.63	1.56
A5	7.11	7.59	8.56	6.47	8.11	8.93
A6	1.47	1.60	2.57	0.79	2.39	3.44
A7	2.49	2.78	2.77	3.19	1.60	0.50
A8	2.02	2.96	3.95	1.78	2.83	3.39
A9	3.77	4.40	4.59	4.39	3.18	2.20

**Table 2:** Available medical resources and staff at medical facilities per day.

Hospital	Available Medical Resources and Staff			
	Non-ICU Beds	ICU Beds	No. of Doctors	No. of Nurses
H1	16	8	4	8
H2	14	6	4	8
H3	17	4	4	8
H4	5	0	1	2
H5	8	0	1	2
H6	6	0	1	2

**Table 3:** Estimated demand based on admissions from week 1 to week 3 of a dengue fever epidemic in Ciudad del Este, Paraguay.

Day		Week 1							Week 2							Week 3						
		1	2	3	4	5	6	7	8	9	10	11	12	13	14	15	16	17	18	19	20	21
P1	A1	0	0	1	1	1	1	1	3	3	3	3	3	3	3	3	3	4	4	4	4	4
	A2	2	2	2	2	3	3	3	3	3	3	3	3	3	3	3	4	4	4	5	5	5
	A3	3	3	3	3	3	3	3	3	3	3	3	3	3	4	4	4	4	4	4	5	5
	A4	2	2	2	2	2	3	3	2	2	2	2	2	2	2	2	2	2	2	3	3	3
	A5	1	1	2	2	2	2	2	3	3	3	3	3	3	4	4	4	4	4	5	5	5
	A6	3	3	3	3	3	3	3	2	2	2	2	2	2	2	2	3	3	3	4	4	4
	A7	2	2	2	2	2	2	2	1	1	1	1	1	1	2	2	2	2	2	2	3	3
	A8	1	1	1	1	1	1	1	1	1	1	1	1	1	1	1	1	2	2	2	2	2
	A9	0	0	1	1	1	1	1	0	0	0	0	0	0	0	0	0	0	0	0	1	1
P2	A1	0	0	0	0	0	0	0	0	0	0	0	0	1	1	1	1	1	1	1	1	1
	A2	0	0	0	0	0	0	0	1	1	1	1	1	1	1	1	1	1	1	1	2	2
	A3	0	0	0	1	1	1	1	0	0	0	0	0	1	1	1	1	1	1	1	1	1
	A4	0	0	0	0	0	0	0	0	0	0	0	0	0	0	0	0	0	1	1	1	1
	A5	0	0	0	0	0	0	0	1	1	1	1	1	1	1	1	1	1	1	1	1	1
	A6	0	0	0	0	1	1	1	0	0	0	0	0	0	1	1	1	1	1	1	1	1
	A7	0	0	0	0	0	0	0	0	0	0	0	0	0	0	0	0	0	0	1	1	1
	A8	0	0	0	0	0	0	0	0	0	0	0	0	0	0	0	0	0	0	0	0	0
	A9	0	0	0	0	0	0	0	1	1	1	1	1	1	2	2	3	3	3	3	3	3

**Table 4:** Estimated demand based on admissions from week 4 to week 6 of a dengue fever epidemic in Ciudad del Este, Paraguay.

Day		Week 4							Week 5							Week 6						
		22	23	24	25	26	27	28	29	30	31	32	33	34	35	36	37	38	39	40	41	42
P1	A1	5	5	4	4	4	4	3	3	3	3	2	2	2	2	2	2	2	2	2	1	1
	A2	5	5	5	5	5	5	4	4	4	4	3	3	3	3	3	3	3	2	2	2	2
	A3	5	5	5	4	4	4	4	4	4	3	3	3	3	3	3	3	2	2	2	1	1
	A4	3	3	3	3	3	2	2	2	2	2	2	1	1	1	1	1	1	0	0	0	0
	A5	5	5	5	4	4	4	3	3	3	3	2	2	2	2	2	2	2	2	1	1	1
	A6	4	4	4	3	3	3	3	3	3	3	2	2	2	2	2	2	2	1	1	1	1
	A7	3	3	3	2	2	2	2	2	2	2	2	1	1	1	1	1	1	1	1	0	0
	A8	2	2	2	1	1	1	1	1	1	1	1	1	0	0	0	0	0	0	0	0	0
	A9	1	1	1	0	0	0	0	0	0	0	0	0	0	0	0	0	0	0	0	0	0
P2	A1	2	1	1	1	1	1	0	0	0	0	0	0	0	0	0	0	0	0	0	0	0
	A2	2	2	2	1	1	1	1	1	1	1	1	1	1	1	1	1	0	0	0	0	0
	A3	1	1	1	1	0	0	0	0	0	0	0	0	0	0	0	0	0	0	0	0	0
	A4	1	1	1	0	0	0	0	0	0	0	0	0	0	0	0	0	0	0	0	0	0
	A5	1	1	1	1	1	1	1	1	1	1	1	0	0	0	0	0	0	0	0	0	0
	A6	1	1	1	0	0	0	0	0	0	0	0	0	0	0	0	0	0	0	0	0	0
	A7	1	0	0	0	0	0	0	0	0	0	0	0	0	0	0	0	0	0	0	0	0
	A8	0	0	0	0	0	0	0	0	0	0	0	0	0	0	0	0	0	0	0	0	0
	A9	3	3	3	3	3	2	2	2	2	1	1	1	1	1	1	1	1	1	1	1	1

**Table 5:** Allocation of all types of patients during week 1 using Model 1.

Types of Patients	Area	Day						
		1	2	3	4	5	6	7
P1	A1	-	-	2	2	2	2	2
	A2	3	3	3	3	3	3	3
	A3	2, 3	2, 3	2, 3	2, 3	2	2	2
	A4	3	3	3	3	3	3	3
	A5	4	1	1	4	1	1	4
	A6	4	1	1	4	1	1	4
	A7	6	6	6	6	6	6	6
	A8	4	1	1	1	1	1	1
	A9	-	-	5	5	5	5	5
P2	A3	-	-	-	3	3	3	3
	A6	-	-	-	-	1	1	1

- no demand for the day.

**Table 6:** Allocation of all types of patients during week 3 using Model 2.

Types of Patients	Area	Day						
		15	16	17	18	19	20	21
P1	A1	2	7	7	7	7	7	7
	A2	3	3, 7	2, 3	2, 3	7	7	7
	A3	2, 3	2	2	2	2, 3	2, 3	2
	A4	3, 5	3, 5	3	3, 5	3,5	3	3
	A5	1	7	7	7	7	7	7
	A6	1, 2	1, 4	4	1	1, 2, 4	2, 4	1, 4
	A7	6	5, 6	5, 6	5, 6	5, 6	5, 6	5, 6
	A8	1	1	1	1	1	1	1
	A9	5	5	1	1, 5	7	7	5
P2	A1	-	-	-	-	-	7	7
	A2	7	7	7	7	7	7	7
	A3	7	7	7	7	7	2, 7	2, 3
	A4	2	2	2	2	3	3	3
	A5	-	-	-	7	7	7	7
	A6	1	1	1	1	2	1	1
	A7	1	7	7	7	7	7	7
	A8	-	-	-	-	1	1	1

- no demand for the day.

**Table 7:** Medical resource shortage during week 3.

Medical Resource	Day						
	15	16	17	18	19	20	21
R1 (non-ICU beds)		−6	−11	−16	−22	−28	−34
R2 (ICU beds)	−2	−5	−8	−12	−15	−19	−23

**Table 8:** Allocation of additional resources for week 3.

Medical Resource	Hospital						Total
	H1	H2	H3	H4	H5	H6	
R1 (non-ICU beds)	0	0	1	20	0	13	34
R2 (ICU beds)	0	0	6	7	0	10	23

## Data Availability

Not applicable.

## References

[1] Guzman M.G., Harris E. (2015). Dengue. Lancet.

[2] Ng C.W.K., Tai P.Y., Mohamed S.O. (2018). Dengue maculopathy associated with choroidopathy and pseudohypopyon: A case series. Ocul. Immunol. Inflamm..

[3] World Health Organization (2018). Dengue and Severe Dengue. https://www.who.int/news-room/fact-sheets/detail/dengue-and-severe-dengue.

[4] Pollett S., Melendrez M.C., Maljkovic Berry I., Duchene S., Salje H., Cummings D.A.T., Jarman R.G. (2018). Understanding dengue virus evolution to support epidemic surveillance and counter-measure development. Infect. Genet. Evol..

[5] Beatty M.E., Stone A., Fitzsimons D.W., Hanna J.N., Lam S.K., Vong S., Guzman M.G., Mendez-Galvan J.F., Halstead S.B., Letson G.W. (2010). Best practices in dengue surveillance: A report from the Asia-Pacific and Americas Dengue Prevention Boards. PLoS Negl. Trop. Dis..

[6] Shepard D.S., Coudeville L., Halasa Y.A., Zambrano B., Dayan G.H. (2011). Economic impact of dengue illness in the Americas. Am. J. Trop. Med. Hyg..

[7] Luh D.L., Liu C.C., Luo Y.R., Chen S.C. (2018). Economic cost and burden of dengue during epidemics and non-epidemic years in Taiwan. J. Infect. Public Health.

[8] Burdett R.L., Kozan E., Sinnott M., Cook D., Tian Y.-C. (2017). A mixed integer linear programing approach to perform hospital capacity assessments. Expert Syst. Appl..

[9] Zhou L., Geng N., Jiang Z., Wang X. (2018). Multi-objective capacity allocation of hospital wards combining revenue and equity. Omega.

[10] Molina-Pariente J.M., Fernandez-Viagas V., Framinan J.M. (2015). Integrated operating room planning and scheduling problem with assistant surgeon dependent surgery durations. Comput. Ind. Eng..

[11] Beroule B., Grunder O., Barakat O., Aujoulat O., Lustig H. (2016). Operating room scheduling including medical devices sterilization: Towards a transverse logistic. IFAC-PapersOnLine.

[12] Steiner M.T.A., Datta D., Steiner Neto P.J., Scarpin C.T., Rui Figueira J. (2015). Multi-objective optimization in partitioning the healthcare system of Parana State in Brazil. Omega.

[13] Heshmat M., Nakata K., Eltawil A. (2018). Solving the patient appointment scheduling problem in outpatient chemotherapy clinics using clustering and mathematical programming. Comput. Ind. Eng..

[14] Shahriari M., Bozorgi-Amiri A., Tavakoli S., Yousefi-Babadi A. (2017). Bi-objective approach for placing ground and air ambulance base and helipad locations in order to optimize EMS response. Am. J. Emerg. Med..

[15] Anparasan A., Lejeune M. (2017). Resource deployment and donation allocation for epidemic outbreaks. Ann. Oper. Res..

[16] Devi Y., Patra S., Singh S.P. (2021). A location-allocation model for influenza pandemic outbreaks: A case study in India. Oper. Manag. Res..

[17] EsraBüyüktahtakın İ.E., Des-Bordes E., Kıbış E.Y. (2018). A new epidemics-logistics model: Insights into controlling the Ebola virus disease in West Africa. Eur. J. Oper. Res..

[18] Liu M., Xu X., Cao J., Zhang D. (2000). Integrated planning for public health emergencies: A modified model for controlling H1N1 pandemic. J. Oper. Res. Soc..

[19] Hernández-Pérez L.G., Ponce-Ortega J.M. (2021). Multi-objective optimization approach based on deterministic and metaheuristic techniques to resource management in health crisis scenarios under uncertainty. Process. Integr. Optim. Sustain..

[20] Mehrotra S., Rahimian H., Barah M., Luo F., Schantz K. (2020). A model of supply-chain decisions for resource sharing with an application to ventilator allocation to combat COVID-19. Nav. Res. Logist..

[21] Lacasa L., Challen R., Brooks-Pollock E., Danon L. (2020). A flexible method for optimising sharing of healthcare resources and demand in the context of the COVID-19 pandemic. PLoS ONE.

[22] Frej E.A., Roselli L.R.P., Ferreira R.J.P., Alberti A.R., de Almeida A.T. (2021). Decision model for allocation of intensive care unit beds for suspected COVID-19 patients under scarce resources. Comput. Math. Methods Med..

[23] Wang C., Deng Y., Yuan Z., Zhang C., Zhang F., Cai Q., Gao C., Kurths J. (2020). How to optimize the supply and allocation of medical emergency resources during public health emergencies. Front. Phys..

[24] Santini A. (2021). Optimising the assignment of swabs and reagent for PCR testing during a viral epidemic. Omega.

[25] Roy S., Dutta R., Ghosh P. (2021). Optimal time-varying vaccine allocation amid pandemics with uncertain immunity ratios. IEEE Access.

[26] Jordan E., Shin D.E., Leekha S., Azarm S. (2021). Optimization in the Context of COVID-19 Prediction and Control: A Literature Review. IEEE Access.

[27] Brown J., Cug J., Kolencik J. (2020). Internet of Things-based Smart Healthcare Systems: Real-Time Patient-Generated Medical Data from Networked Wearable Devices. Am. J. Med. Res..

[28] Moore C. (2020). Medical Internet of Things-based Healthcare Systems: Wearable Sensor-based Devices, Patient-Generated Big Data, and Real-Time Clinical Monitoring. Am. J. Med. Res..

[29] Lewis S. (2020). Wearable Internet of Things Healthcare Systems: Smart Biomedical Sensors, Wireless Connected Devices, and Real-Time Patient Monitoring. Am. J. Med. Res..

[30] Wells R., Miklencicova R. (2021). Emotional Fatigue, Elevated Anxiety Symptoms, and Sustained Psychological Distress in Frontline Medical Staff and Nurses Working with COVID-19 Patients. Psychosociol. Issues Hum. Resour. Manag..

[31] Gibson P., Janikova J. (2021). Moral Injury, Psychological Ill-Health, and Severe Stress among COVID-19 Frontline Respiratory and Intensive Care Physicians and Nurses. Psychosociol. Issues Hum. Resour. Manag..

[32] Adams D., Grupac M. (2021). Stress-related Psychiatric Disorders, Clinically Significant Depression, and Elevated Anxiety Symptoms among Medical Personnel Providing Care to COVID-19 Patients. Psychosociol. Issues Hum. Resour. Manag..

[33] Sun L., DePuy G.W., Evans G.W. (2014). Multi-objective optimization models for patient allocation during a pandemic influenza outbreak. Comput. Oper. Res..

[34] Gamhewage G. (2018). Evolutions in global epidemic and pandemic preparedness. Int. J. Infect. Dis..

[35] Lovera D., Araya S., Mesquita M.J., Avalos C., Ledesma S., Arbo A. (2014). Prospective applicability study of the new dengue classification system for clinical management in children. Pediatric Infect. Dis. J..

[36] Lovera D., Martínez-Cuellar C., Galeano F., Amarilla S., Vazquez C., Arbo A. (2019). Clinical manifestations of primary and secondary dengue in Paraguay and its relation to virus serotype. J. Infect. Dev. Ctries..

[37] Dirección General de Estadística E y C (2015). Paraguay—Proyección de la Población por Sexo y Edad, Según Distrito, 2000–2025. https://www.datos.gov.py/dataset/paraguay-proyeccion-de-la-poblacion-por-sexo-y-edad-segun-distrito-2000-2025.

